# Excision of the Superior Maxilla for Epithelioma of the Cheek and Hard Palate

**Published:** 1872-01

**Authors:** 


*Excision of the Superior Maxilla for Epithelioma of the Cheek and Hard Palate.*—Epitheliomatous growths had been removed from the patient's cheek on two previous occasions, twelve and eight months back respectively. The third recurrence was estimated to have been in existence about sixteen days before the operation in question was undertaken. In the middle of the cheek, and in the cicatrix of the former incisions, was an indurated swelling of about the size of a filbert, surmounted by a small sore, which presented the usual epitheliomatus characters. The greater part of the left hard palate was also involved; there was no apparent affection of the adjacant glands. The patient's general health was very good. For the operation, an incision carried from the external angular process of the frontal bone and round the tumour, skirting widely the diseased tissues, and prolonged to the angle of the month, was found amply sufficient. As the patient's breathing gave rise to serious apprehension towards the close of the operation, the floor of the orbit was removed for the sake of expedition; the whole of the left soft palate was also removed. The hæmorrhage was readily controlled by torsion. The wound was brought together by means of carbolized gut sutures and two hare-lip pins at the points of greatest tension. The patient was at first troubled with monocular diplopia, probably from loss of the attachment of the inferior oblique muscle. He made an excellent recovery.

